# Neural Correlates of Alexithymia Based on Electroencephalogram (EEG)—A Mechanistic Review

**DOI:** 10.3390/jcm14061895

**Published:** 2025-03-11

**Authors:** James Chmiel, Paula Wiażewicz-Wójtowicz, Marta Stępień-Słodkowska

**Affiliations:** 1 Faculty of Physical Culture and Health, Institute of Physical Culture Sciences, University of Szczecin, Al. Piastów 40B blok 6, 71-065 Szczecin, Poland; 2 Doctoral School, University of Szczecin, Mickiewicza 16, 70-384 Szczecin, Poland; 3 Institute of Pedagogy, University of Szczecin, ul. Ogińskiego 16/17, 71-415 Szczecin, Poland

**Keywords:** alexithymia, EEG, electroencephalogram, electroencephalography, QEEG, oscillations, neurophysiology, electrophysiology, neural correlates

## Abstract

**Introduction:** Alexithymia is a multidimensional construct characterized by difficulties in identifying and describing emotions, distinguishing emotional states from bodily sensations, and an externally oriented thinking style. Although the prevalence in the general population is around 10%, it is significantly higher in clinical groups, including those with autism spectrum disorders, depression, anxiety, and neurological conditions. Neuroimaging research, especially using magnetic resonance imaging, has documented structural and functional alterations in alexithymia; however, electroencephalography (EEG)—an older yet temporally precise method—remains less comprehensively explored. This mechanistic review aims to synthesize EEG-based evidence of the neural correlates of alexithymia and to propose potential neurophysiological mechanisms underpinning its affective and cognitive dimensions. **Methods:** A thorough literature search was conducted in December 2024 across PubMed/Medline, ResearchGate, Google Scholar, and Cochrane using combined keywords (“EEG”, “QEEG”, “electroencephalography”, “alexithymia”) to identify English-language clinical trials or case studies published from January 1980 to December 2024. Two reviewers independently screened the titles and abstracts, followed by a full-text review. Studies were included if they specifically examined EEG activity in participants with alexithymia. Of the 1021 initial records, eight studies fulfilled the inclusion criteria. **Results:** Across the reviewed studies, individuals with alexithymia consistently demonstrated right-hemisphere dominance in EEG power and connectivity, particularly in the theta and alpha bands, during both neutral and emotion-eliciting tasks. Many exhibited reduced interhemispheric coherence and disrupted connectivity in the frontal and parietal regions, potentially contributing to difficulties in cognitive processing and emotion labeling. Some studies have also reported diminished gamma band activity and phase synchrony in response to negative stimuli, suggesting impaired higher-order integration of emotional information. Crucially, subjective reports (e.g., valence ratings) often do not differ between alexithymic and non-alexithymic groups, highlighting that EEG measures may capture subtle emotional processing deficits not reflected in self-reports. **Conclusions:** EEG findings emphasize that alexithymia involves specific disruptions in cortical activation and network-level coordination, rather than merely the absence of emotional experiences. Right-hemisphere over-reliance, reduced interhemispheric transfer, and atypical oscillatory patterns in the alpha, theta, and gamma bands appear to be central to the condition’s pathophysiology. Understanding these neural signatures offers avenues for future research—particularly intervention studies that test whether modulating these EEG patterns can improve emotional awareness and expression. These insights underscore the potential clinical utility of EEG as a sensitive tool for detecting and tracking alexithymic traits in both research and therapeutic contexts.

## 1. Introduction

Alexithymia is a multidimensional psychological construct characterized by difficulties in identifying and describing one’s emotions, distinguishing emotions from bodily sensations, and engaging in introspective or imaginative processes [1]. It is often described as an externally oriented thinking style that lacks emotional awareness [2]. This condition is frequently conceptualized in terms of two key dimensions: a cognitive dimension, involving challenges in recognizing, interpreting, and articulating emotions [3,4], and an affective dimension, centered on difficulties in experiencing emotional arousal and engaging in imaginative or introspective thought [5]. Approximately one in ten individuals in the general population experiences alexithymia, although the prevalence varies based on factors such as sex, age, culture, education, and socioeconomic status [6,7,8]. Rates are considerably higher in clinical populations, including those with autism (50%) [9], depression (21–42%) [10], schizophrenia (35%) [11], eating disorders (40–63%) [12], substance use disorders (42–67%) [13], and anxiety disorders (13–27%) [14]. It also occurs frequently in neurological conditions such as traumatic brain injury [15], stroke [16], epilepsy [17], Parkinson’s disease [18], and Alzheimer’s disease [19].

In non-clinical populations, alexithymia is relatively stable over time, particularly in adulthood, reinforcing its conceptualization as a trait [20]. Nevertheless, variability exists, with younger women often reporting lower prevalence rates than older individuals or men [21]. This stability highlights the profound psychological and social implications of alexithymia. Individuals who struggle to recognize and communicate emotions face heightened susceptibility to stress, anxiety, and depression [22], along with interpersonal difficulties that can contribute to social isolation [23].

Treatments for alexithymia aim to enhance emotional awareness, facilitate expression, and improve overall psychosocial functioning of patients. Mindfulness-based interventions (e.g., Mindfulness-Based Stress Reduction and Mindfulness-Based Cognitive Therapy) show moderate effectiveness in reducing alexithymia symptoms over three to nine months [24]. Cognitive Behavioral Therapy (CBT) also remains a core approach, with Acceptance and Commitment Therapy (ACT), in particular, emphasizing psychological flexibility to achieve more robust outcomes [25].

To understand the brain mechanisms of alexithymia, neuroimaging methods are needed. Magnetic resonance imaging (MRI) remains the most commonly employed technique in this context, with numerous reviews documenting altered brain function and structure in individuals with alexithymia. However, the earliest neuroimaging method, electroencephalography (EEG), can also be used as a valuable tool in this context.

EEG captures the brain’s electrical activity by placing sensors on the scalp, allowing scientists and clinicians to monitor large groups of neurons in real time. A key advantage of this technique is its ability to reveal rapid shifts in brain activity measured in milliseconds. This precision makes EEG particularly useful for investigating information processing, responses to stimuli, and neural activity during the different sleep stages. In medical settings, EEG is commonly employed to diagnose and monitor conditions such as epilepsy and sleep disorders. Because the equipment is reasonably priced, easy to transport, and causes no discomfort, EEG remains a versatile choice for both hospitals and research facilities. Insights gained from EEG have significantly advanced our understanding of language, attention, and consciousness and contributed to the development of brain–computer interfaces designed to assist people with severe mobility impairments [26,27,28].

In contemporary EEG research, quantitative EEG (QEEG) techniques are frequently employed to transform raw signals into mathematically derived metrics that can be compared across groups or correlated with clinical measurements. QEEG involves a range of approaches, including power spectral analysis (examining delta, theta, alpha, beta, and gamma bands), coherence measures (assessing functional connectivity between brain regions), and topographic mapping (visualizing scalp distributions of specific frequency bands). These quantitative indices enhance the interpretability of EEG data by providing stable, statistically analyzable parameters that are indicative of the underlying neurophysiological processes. QEEG has contributed to the identification of distinct brain activity patterns in various clinical conditions, aiding in diagnostic clarification and guiding therapeutic approaches [29,30,31,32,33,34]. In the study of alexithymia, QEEG offers an opportunity to pinpoint spectral and connectivity abnormalities that may reflect core difficulties in emotion recognition and processing. Therefore, integrating QEEG metrics in both resting and task-based paradigms could illuminate specific pathophysiological pathways linked to the cognitive and affective components of alexithymia.

This *mechanistic review* focuses on the potential pathophysiological mechanisms underlying alexithymia, as revealed by continuous EEG activity. Specifically, we compile and examine studies employing continuous (non-averaged) EEG recordings—encompassing both resting-state and task-based paradigms—to elucidate trait-like neural signatures. We explicitly exclude event-related potentials (ERPs) and other time-locked methodologies to enable a targeted analysis of broader and ongoing brain activity. By highlighting how EEG patterns may reflect the neurobiological processes underlying alexithymia, this review aims to advance our understanding of its etiology and to inform future research directions.

## 2. Methods

The objective of this mechanistic review is to systematically assess the EEG patterns in alexithymia. To guarantee the validity and applicability of the evidence included, a thorough literature search and strict selection criteria were used. The methodology was partially based on accepted practices for conducting systematic reviews and evidence synthesis (PRISMA), with an emphasis on identifying original empirical studies—including observational, experimental, and case studies—that evaluated the EEG in alexithymia. However, this is a mechanistic review rather than a systematic review, aiming to explain the pathophysiological mechanisms of alexithymia based on EEG findings. As such, it does not follow all of the PRISMA methodology that is commonly used in systematic reviews. This review does not include an assessment of the Risk of Bias and PICOS.

### 2.1. Data Sources and Search Strategy

For this review, J.C., M.S.-S. and P.W.-W. conducted an independent standards-compliant Internet search using the following set of combined keywords: “EEG” OR “QEEG” OR “electroencephalogram” OR “electroencephalography” OR “electrophysiology” OR “electrophysiologic” AND “alexithymia” OR “alexithymic”. A comprehensive search was carried out in December 2024 using several databases: PubMed/Medline, Scopus, Research Gate, Google Scholar, and Cochrane, with an emphasis on publications released between January 1980 and December 2024.

### 2.2. Study Selection Criteria

To be eligible for inclusion in this review, the publications had to be clinical trials or case studies published in English between January 1980 and December 2024, and they specifically examined EEG activity in individuals with alexithymia. Review articles and papers not written in English were excluded from the study.

### 2.3. Screening Process

A structured screening process was implemented to ensure the inclusion of relevant studies and exclusion of those that did not meet predefined criteria. In the first screening step, abstracts and titles were carefully examined by three independent reviewers, J.C., M.S.-S. and P.W.-W.

#### 2.3.1. Title and Abstract Screening

Each reviewer evaluated the abstracts and titles of the independently accessible records to determine which research satisfied the inclusion criteria. The primary emphasis of the screening criteria for alexithymia was EEG.

#### 2.3.2. Full-Text Assessment

Publications that passed the title and abstract screening were subsequently subjected to a detailed full-text evaluation. The reviewers thoroughly examined each article to verify its compliance with the eligibility criteria, with particular emphasis on confirming that the studies were clinical trials or case studies published in English between January 1980 and December 2024.

## 3. Results

**Figure 1:**
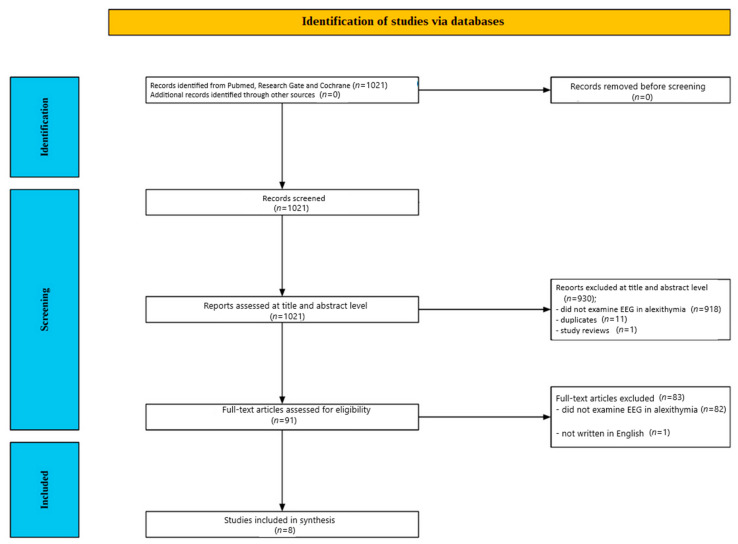
Flow chart depicting the different phases of the systematic review.

Figure 1 illustrates the screening process. Initially, 1021 studies were identified through database searches. Of these, 930 publications were excluded after a review of their abstracts and titles: 918 did not investigate EEG in alexithymia, 11 were duplicates, and one was a review article. Following a detailed full-text review of the remaining 91 papers, 82 studies were excluded for not addressing EEG in alexithymia, and one was excluded because it was written in a language other than English. Ultimately, eight studies met the inclusion criteria after a thorough content evaluation.

The eight included studies [35,36,37,38,39,40,41,42] were published between 2001 and 2017.

### 3.1. Summary of Included Studies

Eight studies (see Table 1 for full details) were included in this review, each of which examined the neural correlates of alexithymia using continuous EEG recordings. Sample sizes ranged from relatively small groups of 20–24 participants [38,42] to larger groups of up to 130 participants [39]. Across all studies, right-handedness was a common inclusion criterion, likely to reduce confounding effects related to hemispheric dominance. Most investigations involved university students or young adult samples, although one study focused on a clinical group with borderline personality disorder [41] and another targeted a cohort of nurses [39].

Alexithymia assessment has consistently relied on variations of the Toronto Alexithymia Scale (TAS), most commonly the TAS-20 [37,38,41,42], although other versions, such as the TAS-26, have also been reported [36]. Cutoff scores for classifying “alexithymic” vs. “non-alexithymic” participants varied across studies, ranging from 51 to 74. One study employed the Bermond-Vorst Alexithymia Questionnaire (BVAQ) [40]. Additional psychological measures (e.g., SCL-90-R, Beck Depression Inventory) were sometimes included to control for depression and general psychopathology [41].

Regarding EEG methodology, all studies used multichannel EEG systems with placements following the 10–20 or 10–10 system, although the number of electrodes ranged from 16 [39] to 62 [35,36]. Frequency band analyses predominantly focus on theta, alpha, beta, and gamma bands, with some studies further subdividing theta or alpha ranges (e.g., alpha-1/alpha-2). Most studies employed spectral power as a primary outcome; some incorporated coherence or phase synchronization measures [38,39,40] or asymmetry indices [35,36,41]. Stimulus paradigms ranged from resting-state protocols [37,41] to emotion-evoking tasks using film clips and images [35,36,38,40,42].

### 3.2. EEG Activity in Patients with Alexithymia

Multiple EEG-based investigations [35,36,37,38,39,40,41,42] consistently show that while individuals with alexithymia often report subjective emotional experiences comparable to non-alexithymic controls, their neural responses during emotion processing are notably different. In studies using film clips to elicit target emotions, the self-reported intensity of emotions (e.g., fear, anger, or erotic stimuli) typically does not differ significantly between alexithymic and control groups [35,36]. However, EEG measures reveal pronounced divergences. One common finding is heightened right-hemisphere involvement—particularly in the alpha-2 and theta frequency ranges—during exposure to emotionally evocative stimuli [35,36]. For instance, alexithymic participants often show alpha-2 desynchronization localized to the right centroparietal cortex, alongside increased bilateral (or right-lateralized) theta synchronization in the anteriotemporal regions for anger- or stress-inducing films. In contrast, controls typically demonstrate more bilateral activation patterns, with left-hemisphere dominance being more frequently observed in non-alexithymic participants.

In addition to alpha- and theta-band differences, gamma activity has emerged as a key marker differentiating individuals with alexithymia and those without alexithymia. Studies have indicated reduced gamma band power and phase synchrony in alexithymic participants when processing negative stimuli [38]. Although both groups may exhibit increased gamma activity for negative compared to neutral images, this increase tends to be more robust and broadly distributed (e.g., centroparietal areas) in non-alexithymic participants, whereas alexithymics show minimal to no gamma band enhancement. Moreover, certain studies have pinpointed the temporal dynamics of theta activity, revealing a delayed theta synchronization peak in individuals with alexithymia (400–600 ms post-stimulus) relative to controls (200–300 ms), as well as a more pronounced right-hemisphere response to unpleasant stimuli [42].

Connectivity and coherence analyses offer further insights into alexithymia-related neural alterations. Research highlights reduced interhemispheric coherence across the frontal and central regions (e.g., F3–F4, C3–C4) and diminished intrahemispheric connectivity between the fronto-occipital and parieto-central sites, predominantly in the left hemisphere [39]. Similarly, in default mode network (DMN) regions—particularly the right anterior cingulate cortex (ACC) and right posterior cingulate cortex (PCC)—alexithymic participants display decreased alpha-band connectivity that correlates negatively with alexithymia severity, even after controlling for general psychopathology [37]. These patterns indicate widespread disruptions in network integration in individuals with alexithymia, manifested as reduced interhemispheric cooperation and selective connectivity deficits in the DMN and other cortical networks involved in emotional processing.

Finally, clinical investigations of borderline personality disorder (BPD) samples underscore the generalizability of these EEG findings. Even though resting-state frontal alpha asymmetry (FAS) does not always distinguish clinical groups from controls, within BPD samples, higher alexithymia scores correlate with right-frontal FAS, highlighting a specific relationship between neural lateralization and emotional awareness deficits [41].

## 4. Discussion

Collectively, these studies provide compelling evidence that alexithymia is associated with distinctive patterns of cortical activation, connectivity, and hemispheric asymmetry during both resting-state and emotion-eliciting conditions. Despite methodological differences—such as variations in sample sizes, emotion-induction paradigms, and EEG frequency band analyses—several convergent findings have emerged that illuminate how individuals with alexithymia differ from controls at the neural, behavioral, and connectivity levels.

Across multiple studies [35,36], alexithymic and non-alexithymic participants did not differ significantly in their self-reported emotional intensity or valence ratings. This convergence suggests that while individuals with alexithymia may experience comparable emotional reactions to stimuli “on-paper”, their brain activity reveals atypical patterns of emotional processing. In studies that included behavioral indices such as reaction times or subjective ratings [38,39], the absence of clear group differences in ratings underscores the subtlety of alexithymic traits and the limitations of self-report in reliably capturing the emotional processing disruptions documented at the neurophysiological level.

A prominent theme in these studies is the heightened role of the right hemisphere in alexithymia. Studies [35,36] show right-hemisphere-dominant activity in individuals with alexithymia, particularly in the theta and alpha bands, when they respond to emotional stimuli. This right-lateralized pattern extends to resting-state conditions as well. For instance, in a study [37], alpha power reductions in the right posterior cingulate cortex (PCC), coupled with decreased functional connectivity in right-lateralized regions, suggest a network-based mechanism of altered emotion processing. Similarly, studies [39,40] show that reduced interhemispheric coherence and disrupted connectivity patterns often involve the right hemisphere, reinforcing the idea that right-hemisphere regions critical for emotional awareness and regulation are either under- or over-engaged in alexithymia.

Many of the included experiments [35,36,42] also reveal distinctive modulations in alpha and theta power during emotional tasks in alexithymic participants. For example, the consistent pattern of alpha desynchronization in the right centroparietal and parietal cortices during both negative and positive film clips [35,36] suggests an altered cortical arousal in response to emotional stimuli. Concurrently, increased theta synchronization—particularly in the anteriotemporal areas—may reflect compensatory or dysregulated processing mechanisms. This combination of alpha desynchronization and theta synchronization in the right hemisphere is a recurring finding and may underlie the challenges that individuals with alexithymia face in decoding and articulating emotional states.

A study [38] provides valuable insight into how alexithymia affects higher-frequency oscillations, illustrating that individuals with alexithymia fail to exhibit the robust increases in gamma power and phase synchrony commonly observed in non-alexithymic participants in response to negative stimuli. Gamma band synchronization is often linked to the higher-order integration of sensory, cognitive, and emotional information. Its reduction in alexithymia signals a possible deficit in the binding processes necessary to form cohesive, emotional representations. Furthermore, reduced gamma phase synchrony across widespread cortical regions indicates weakened communication among neural circuits supporting emotional appraisal, suggesting that individuals with alexithymia may struggle to effectively integrate emotionally relevant cues.

Several studies [37,39,40] converge on the finding that alexithymia is characterized by reduced coherence and altered functional connectivity both between and within hemispheres. In a study [37], diminished connectivity in the alpha and beta bands between key areas of the default mode network (DMN), including the ACC, PCC, and frontal regions, was associated with higher alexithymia scores, independent of general psychopathology. Studies [39,40] reinforce these findings, showing impaired connectivity specifically between the right frontal lobe and the left hemisphere in individuals with alexithymia. The lack of robust interhemispheric integration may underlie the hallmark difficulties that individuals with alexithymia face in identifying and describing feelings. Additionally, lower intrahemispheric coherence in fronto-occipital and parieto-central pathways, observed in these studies, suggests that connectivity disruptions extend beyond emotion-specific networks to reflect broader integrative challenges in coordinating frontal “executive” areas with posterior “perceptual” regions.

Study [41] investigated whether resting-state frontal alpha asymmetry—a measure long associated with emotion and motivational tendencies—could serve as a biomarker for alexithymia in borderline personality disorder (BPD) populations. While no significant group-level differences in FAS scores were found between patients with BPD and healthy controls, a significant positive correlation emerged between the FAS and total TAS-20 scores within the BPD group. Specifically, patients with BPD and higher right frontal alpha activity (interpreted as lower cortical activation in the right frontal lobe) tended to exhibit higher levels of alexithymic traits. The absence of this pattern in healthy controls underscores that the relationship between frontal asymmetry and alexithymia may be context-dependent and potentially amplified by the broader emotional dysregulation observed in BPD.

A study [42] sheds light on the temporal dimension of emotional processing deficits, showing that alexithymic participants exhibit delayed theta synchronization peaks for both pleasant and unpleasant stimuli. Reduced and delayed early theta event-related synchronization (ERS) in the left anterior cortical regions, alongside enhanced right anterior theta synchronization for unpleasant stimuli, reflects disproportionate right-hemisphere engagement. This temporal lag may represent either a compensatory mechanism or a fundamental delay in the integration of emotional information during the early stages of perception.

Taken together, these studies suggest that alexithymia involves disrupted neural connectivity and atypical lateralization rather than the simple absence of emotional experience. Individuals with alexithymia appear to register emotional intensity at a subjective level (as evidenced by similar self-reported emotion ratings) but process these emotions in a neurologically distinct manner. Neurophysiological findings converge on the critical role of right-hemisphere regions, especially the parietal, temporoparietal, and frontal areas, in the generation and integration of emotional information. Repeated observations of reduced coherence and heightened right-sided alpha or theta activity suggest a fundamental disruption in how higher-order cognitive processes (e.g., labeling and interpretation) are integrated with “bottom-up” emotional signals.

The collective results underscore that alexithymia is not merely a behavioral phenomenon characterized by a diminished emotional vocabulary but is grounded in measurable alterations in neural activity and neural connectivity. Future research should aim to refine our understanding of how these cortical patterns evolve over time and whether therapeutic interventions targeting interhemispheric coordination or strengthening connectivity in right-lateralized emotional networks can alleviate the core deficits of alexithymia. Longitudinal and intervention studies could clarify whether these neurophysiological markers serve as endophenotypes—stable traits that predispose individuals to alexithymic difficulties—or whether they are amenable to change through targeted therapy (e.g., neuromodulation, mindfulness, or emotion-focused interventions). Ultimately, understanding these neural underpinnings may improve diagnostic specificity and foster novel treatment strategies for individuals with alexithymia across diverse clinical contexts.

## 5. Mechanisms of EEG Patterns in Alexithymia

**Figure 2:**
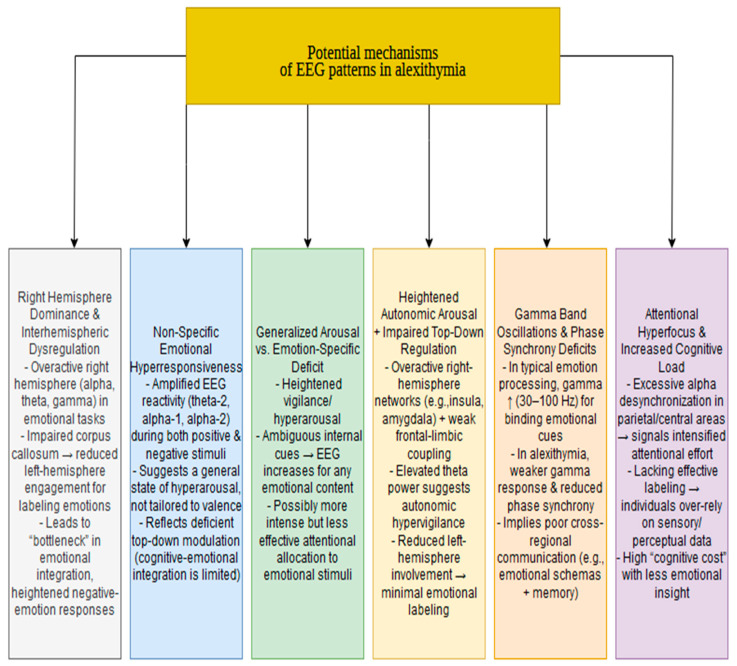
Potential mechanisms of EEG patterns in alexithymia.

The studies reviewed provide a detailed account of the EEG differences between individuals with alexithymia and healthy controls. Although EEG is a powerful diagnostic tool with high temporal resolution, it provides indirect insights into the underlying mechanisms of pathophysiological differences. This section proposes theoretical explanations for the observed patterns, acknowledging that further research is necessary to validate or refute these hypotheses. The proposed mechanisms are shown in Figure 2.

### 5.1. Right-Hemisphere Dominance and Interhemispheric Dysregulation

The right hemisphere plays a central role in processing emotional stimuli, particularly those involving nonverbal and negative emotional cues [43,44]. EEG studies consistently report heightened activity in the right hemisphere of individuals with alexithymia across various EEG frequency bands, including alpha, theta, and gamma bands. This suggests an over-reliance or dysregulation of right-hemisphere networks in emotional processing. Heightened alpha power in the right centroparietal and parietal regions was observed during both neutral and emotional conditions, indicating cortical inhibition in individuals with alexithymia. This suppression may reflect a neural bottleneck, where the right hemisphere struggles to manage emotional stimuli effectively, thereby reducing the capacity for cognitive-emotional integration.

In addition to alpha activity, increased theta synchronization in the right hemisphere, particularly in the anterior regions, is frequently observed during negative emotional processing. This pattern aligns with evidence suggesting that theta synchronization may represent compensatory activity to manage emotional overload [45]. The right hemisphere’s heightened sensitivity to negative emotions appears to overwhelm the system, inhibiting coherent emotional integration and contributing to the inability to interpret or articulate feelings effectively. This dominance of the right hemisphere is often coupled with impaired interhemispheric communication [46]. Disruptions in corpus callosum function exacerbate right-hemisphere dominance by preventing the balanced engagement of the left hemisphere, which is crucial for verbalizing and cognitively processing emotions. Dysregulation of specific cortical areas, such as the anterior insula, supports this mechanism. The anterior insula, a critical node in the salience network associated with the right hemisphere, integrates interoceptive signals and emotional salience but appears hyperactive or dysregulated in individuals with alexithymia [47].

The dominance of the right hemisphere in alexithymia is likely to result from a combination of neural inefficiency. Overcompensation occurs when the right hemisphere attempts to handle emotional processing demands that are typically distributed across both hemispheres, leading to overload and inefficiency. Simultaneously, poor interhemispheric transfer reduces the left hemisphere’s engagement, preventing it from contributing to the higher-order processing needed for emotional labeling and articulation. This functional imbalance hinders dynamic emotional valence processing, particularly for complex or positive emotional stimuli.

EEG findings of increased alpha and theta activity in the right hemisphere during emotional tasks align with this model of right-hemisphere dominance. These patterns reflect heightened cortical arousal or compensatory efforts in the right hemisphere, indicative of individuals with alexithymia’s difficulties in effectively managing emotional stimuli. This dysregulation likely underpins the core challenges of emotional awareness and articulation that characterize alexithymia.

### 5.2. Non-Specific Emotional Hyper-Responsiveness

Alexithymia, defined as an impairment in recognizing, differentiating, and verbally expressing emotions, is often associated with atypical patterns of neural activation during emotional processing [48,49]. In the included studies, EEG recordings were analyzed in alexithymic and control participants as they viewed emotional video clips designed to elicit both positive (joy) and negative (anger, fear, and stress) states. One of the main observations was increased EEG reactivity in the theta-2, alpha-1, and alpha-2 frequency bands in the anterior and posterior regions of the right hemisphere in individuals with alexithymia. This heightened activity occurred irrespective of whether the stimulus was positive or negative, suggesting a non-specific amplification of the emotional reactivity. Right-hemisphere hyper-responsiveness has been documented in individuals with alexithymia, potentially reflecting altered cognitive-emotional integration [48,50]. These findings align with the notion that alexithymic traits may predispose individuals to intensify or misconstrue emotional signals due to impaired top-down affect regulation [49,51].

Additionally, alexithymic participants displayed pronounced desynchronization in alpha bands (alpha-1, alpha-2) in the parietal and centroparietal areas in response to fear and stress, whereas control participants showed more bilateral (i.e., balanced) alpha-band desynchronization. Alpha desynchronization (i.e., a reduction in alpha power) is typically associated with increased cortical arousal and attentional demands [52]. In alexithymia, exaggerated desynchronization in the parietal regions could signify amplified attentional engagement with emotional stimuli without appropriate affect labeling and regulation [49]. This may reflect a compensatory mechanism in which individuals with alexithymia attempt to process emotional content but lack the usual top-down modulation that facilitates efficient emotional appraisal [53].

Taken together, these findings highlight that individuals with alexithymia exhibit a general pattern of heightened physiological reactivity to emotional stimuli. Rather than selectively modulating their responses based on positive or negative valence, individuals with alexithymia demonstrate overall intensified and somewhat dysregulated neural engagement, possibly reflecting core deficits in identifying and contextualizing their affective experiences.

### 5.3. Generalized Arousal Versus Emotion-Specific Deficits

One possible mechanism is that the observed EEG changes in alexithymia reflect a generalized arousal response rather than a deficit in processing specific emotions. In this framework, heightened reactivity, particularly in the right hemisphere, may indicate increased vigilance or hyperarousal. Instead of failing to process emotions, individuals with alexithymia may experience ambiguous internal cues that they find difficult to label. This mismatch between high arousal and deficient labeling could manifest as non-specific increases in cortical activation for both positive and negative stimuli [54,55].

EEG patterns may also indicate abnormal attentional allocation to emotional stimuli, where individuals with alexithymia focus more intently—albeit ineffectively—on emotional cues, driving increased cortical engagement in the parietal and centroparietal regions. From a cognitive load perspective, the difficulty in identifying and labeling emotions may consume additional resources, leading to more pronounced desynchronization in alpha bands (a known marker of increased attentional or working memory demands) [52,56].

From a developmental perspective, differences in early life emotional socialization may shape the functional organization of cortical networks responsible for emotional awareness and expression. These variations could yield a neural profile characterized by less effective top-down regulation via the prefrontal cortex and greater bottom-up reactivity to emotionally salient stimuli (i.e., right hemisphere activation). Such a pattern could appear on EEG as the heightened or non-specific reactivity observed in alexithymic participants [48,57].

Comorbid conditions commonly associated with alexithymia, such as anxiety, depression, and somatic complaints, may further amplify EEG reactivity [58]. Heightened responses may thus partially reflect affective instability or dysregulation stemming from these overlapping conditions. For instance, fear and stress triggers could be amplified by an underlying anxious predisposition, leading to more pronounced alpha desynchronization and right-hemisphere activation. Moreover, another hallmark of alexithymia is an externally oriented thinking style, wherein individuals focus on external details rather than internal emotional states. This could drive atypical cortical engagement: while typical populations might show balanced or bilateral alpha desynchronization (reflecting both self-referential and external processing), alexithymics might over-rely on parietal regions (sensory processing) and under-rely on frontal networks (reflection and mentalization). The net effect is a lopsided or exaggerated cortical pattern in EEG [49,59].

### 5.4. Heightened Autonomic Arousal with Impaired Top-Down Regulation

A key explanatory framework posits that individuals with alexithymia experience heightened autonomic arousal but struggle to label or downregulate these bodily and emotional signals [60]. This imbalance may lead to non-specific patterns of EEG activation—including increased power in theta-2, alpha-1, and alpha-2 bands—regardless of whether the stimuli are positive or negative.

Empirical work using both fMRI and EEG techniques consistently shows that individuals with alexithymia display more pronounced right-hemisphere engagement in response to emotional stimuli [61]. One interpretation is that the right hemisphere’s dominance in processing emotional and bodily signals is exacerbated in individuals with alexithymia owing to the weaker linguistic or executive control typically associated with the left hemisphere and medial prefrontal regions [54]. As a result, the brain over-responds to emotional triggers in a diffuse manner rather than selectively modulating its response in line with the emotional valence of the stimuli.

From a neurophysiological perspective, the amygdala and insula play central roles in alerting the cortex to salient emotional and interoceptive signals [62]. In healthy individuals, the prefrontal cortex (particularly the ventromedial and dorsolateral regions) integrates and shapes these inputs, enabling the labeling, context-based interpretation, and regulation of emotional reactions [63]. In alexithymia, this top-down modulation appears attenuated, leaving a relatively uninhibited or “raw” emotional signal that is registered as heightened autonomic arousal but is not effectively named, differentiated, or suppressed [48].

Studies on EEG frequency bands lend further weight to this theory. For instance, elevated theta activity (particularly in the theta-2 range) has been linked to intensified emotional processing and autonomic hypervigilance [64]. Meanwhile, abnormalities in alpha bands (alpha-1 and alpha-2) can signify difficulties with cortical inhibition and focused attention, indicating unsuccessful top-down filtering of emotional input [46].

### 5.5. Gamma Band Oscillations and Phase Synchrony Deficits

Study [38] investigated gamma band activity and synchronization in individuals with alexithymia while processing emotional stimuli. Gamma oscillations (approximately 30–100 Hz) have long been recognized as integral to feature binding, attention, and memory retrieval [65,66]. When emotionally salient stimuli—especially negatively valenced or threatening images—are presented, human brains often exhibit heightened gamma band power and phase synchronization, reflecting intensive communication across distributed neural networks [67,68]. These patterns underscore the role of gamma oscillations in integrating sensory signals with emotional memory and appraisal processes, thereby facilitating adaptive responses.

In a study [38], non-alexithymic participants showed increased gamma band power and phase synchronization in the 400–450 ms time window following negative emotional stimuli. This temporal window is widely regarded as crucial for cognitive-emotional evaluation and working memory recruitment [69], suggesting that the observed gamma enhancement signifies the efficient integration of emotional cues with existing memory structures. Such integration also engages top-down modulatory pathways that likely encompass semantic and autobiographical schemas.

In contrast, other studies did not display this increase in gamma activity, a finding that implies two complementary mechanisms. First, deficient phase synchronization points to weakened cross-regional neuronal coherence, which is essential for coherent emotional appraisal [70]. Second, the absence of robust gamma oscillatory recruitment may signal a failure to activate relevant emotional schemas, aligning with the central features of alexithymia—specifically the inability to identify and describe internal affective states. The lack of gamma-based network integration thus becomes a neural hallmark of the attenuated emotional responses observed in alexithymia.

More broadly, alexithymia is associated with impoverished emotional awareness, limited introspection, and reduced emotional lexicon. The absence of a gamma response observed here further hints at the under-recruitment of memory and conceptual networks that typically support emotional categorization and self-reflection.

### 5.6. Attentional Hyperfocus and Increased Cognitive Load

One explanatory model attributes the altered EEG patterns in individuals with alexithymia—particularly the increased alpha desynchronization in the parietal and central-parietal regions—to attentional hyperfocus and elevated cognitive load during emotional processing. Despite their difficulties in labeling emotions, individuals with alexithymia may, as a compensatory strategy, allocate excessive cognitive resources to the sensory and perceptual aspects of emotional cues. This over-engagement leads to atypically high levels of cortical activation.

In typical emotion processing, well-coordinated top-down mechanisms, such as prefrontal-limbic coordination, enable the efficient interpretation and categorization of affective stimuli [56]. However, in alexithymia, the weakness or absence of emotional labeling strategies forces individuals to rely on external signals, leading to intensified attention and cognitive effort.

Alpha power reduction (i.e., alpha desynchronization) is a hallmark of increased cortical workload. Research shows that alpha desynchronization, especially in the parietal and central-parietal areas, correlates with heightened attentional demands and cognitive effort [46]. In alexithymia, this stronger desynchronization likely reflects an attempt to interpret ambiguous or poorly labeled emotional content by focusing on external cues. Consequently, limited interoceptive abilities (awareness of internal bodily states) shift attention outward, depleting cognitive resources and prolonging alpha wave desynchronization [49]. Despite their increased mental effort, individuals with alexithymia often fail to achieve clear internal emotional insights or effective emotional labeling [51].

## 6. Limitations and Future Directions

### 6.1. Standardization of EEG Protocols

Although EEG studies on alexithymia have yielded valuable findings, the field remains hampered by methodological inconsistencies that complicate cross-study comparisons [71]. One major issue is the variability in frequency band definitions; while some researchers define the alpha band as 8–12 Hz, others extend it to 8–13 Hz, making it difficult to ascertain whether observed differences truly reflect underlying neural mechanisms or arise from methodological choices [52]. Electrode placement and reference also vary considerably. While some studies rely on minimal-channel systems or linked-ear references, others use high-density arrays (e.g., 64+ electrodes) and average-reference. This heterogeneity can obscure genuine topographical patterns linked to alexithymia by introducing confounding factors into spatial resolution.

Preprocessing approaches similarly diverge: some investigators employ strict artifact-rejection strategies, whereas others use Independent Component Analysis (ICA) to correct ocular or muscle artifacts [72,73]. Such variability can significantly influence the reported power changes, particularly in the alpha and theta bands. Moreover, filtering ranges differ across laboratories, raising the question of whether the observed shifts in oscillatory activity reflect true neurophysiological differences or merely stem from disparate filtering practices.

Additionally, emotion-elicitation paradigms are not uniform. Studies have employed film clips, static pictures (e.g., the International Affective Picture System, IAPS), and other stimuli to provoke emotional states [74]. While methodological diversity can illuminate a broader spectrum of emotional processes, it hinders direct comparisons and meta-analyses. Researchers have therefore called for a standardized core set of validated tasks and consistent neutral baselines to ensure the comparability of EEG outcomes. Enhanced transparency in reporting artifact thresholds, epoch lengths, frequency bandwidths, and effect sizes—along with multi-site collaborations under pre-established protocols—could substantially reduce between-study variability [71]. Such unified efforts would help establish the best practices for EEG investigations in alexithymia, ultimately improving replicability and clinical relevance.

### 6.2. Larger, More Diverse Samples

A major challenge in alexithymia research is the reliance on small, homogeneous samples, often drawn from undergraduate students or narrowly defined clinical groups [75]. While these cohorts provide valuable initial insights, they limit the generalizability of EEG findings. Future studies should recruit larger and more diverse samples spanning different demographic, cultural, and clinical backgrounds to strengthen external validity [76].

Expanding sample sizes across multiple recruitment sites is particularly important for detecting moderate or subtle EEG effects that may be overlooked in smaller studies. Multi-lab collaborations and data pooling can increase statistical power, improving our ability to identify relationships between specific EEG markers (e.g., alpha asymmetry and theta coherence) and varying degrees of alexithymic symptoms. Additionally, including underrepresented groups—such as older adults, adolescents, and non-Western populations—can clarify whether EEG signatures of alexithymia (e.g., right-hemisphere overactivation, decreased interhemispheric connectivity) remain consistent across age groups and cultural norms [77,78].

Greater sample diversity also allows subgroup analyses, distinguishing between individuals with primary alexithymia (trait-based, independent of other diagnoses) and those with secondary alexithymia linked to psychiatric conditions like depression or anxiety. Identifying EEG patterns specific to alexithymia, rather than general comorbidities, could refine the diagnostic criteria and subtype classification [79].

Cross-cultural studies could further determine whether EEG patterns reflect the biological underpinnings of alexithymia or cultural variations in emotional expression [80]. For example, externally oriented thinking or emotional suppression may influence alpha and theta activity, independent of core alexithymic traits [81]. Establishing cross-culturally validated EEG norms would help distinguish genuine neurophysiological deviations from artifacts introduced by sampling bias.

Recruiting larger, demographically diverse samples will not only enhance replicability and statistical power but also provide deeper insights into how cultural, developmental, and clinical factors shape neural correlates of alexithymia.

### 6.3. Longitudinal and Developmental Studies

Most research on alexithymia is cross-sectional, making it difficult to determine whether atypical EEG patterns precede or result from emotion-processing deficits. Longitudinal studies tracking individuals from childhood to adulthood could clarify whether these neural signatures emerge early, remain stable, or change in response to environmental factors, such as social support or targeted interventions.

Identifying critical periods for intervention is equally important. If right-hemisphere hyperactivation or reduced interhemispheric coherence is present in adolescence and remains stable, early interventions may help retrain maladaptive neural patterns before they become established. Conversely, if EEG anomalies worsen during key developmental stages (e.g., adolescence and early adulthood), screening and prevention efforts should focus on these periods.

A longitudinal approach would also track the natural fluctuations in alexithymic traits. Some individuals may show spontaneous improvement, possibly due to increased emotional awareness or changes in social contexts [82]. Examining EEG correlates of these changes could identify neural predictors of improvement versus persistent deficits. Additionally, studying EEG in relation to cognitive and social milestones—such as perspective-taking ability or stable interpersonal relationships—could offer a deeper understanding of how alexithymia evolves within the broader psychosocial development.

Finally, repeated assessments of mental health, personality, and environmental factors could help disentangle the effects of comorbid conditions like anxiety or depression. For instance, if right frontal alpha asymmetry predicts worsening anxiety in late adolescence [83], this could indicate a critical window for targeted interventions. Integrating EEG with multi-domain assessments would enable researchers to unravel causal pathways and refine evidence-based interventions that prevent alexithymic patterns from becoming chronic.

### 6.4. Multimodal Imaging and Network Analyses

While EEG provides high temporal resolution for studying emotional processing in alexithymia, its limited spatial precision makes it difficult to pinpoint the brain regions involved in this process. Combining EEG with other neuroimaging modalities—such as fMRI, PET, NIRS, or MEG—can help overcome this limitation and enable more robust network-level analyses [84]. For example, simultaneous EEG-fMRI allows researchers to map transient changes in alpha or theta power onto BOLD signals in the insula, anterior cingulate cortex, or amygdala, clarifying whether cortical oscillations align with over- or underactivation in limbic circuits [85,86,87]. Source localization techniques (e.g., LORETA, sLORETA, beamforming) enriched with MRI data can further refine these findings and identify whether interhemispheric dysregulation stems from frontal callosal pathways or posterior parietal connections [88].

Integrating psychophysiological and neuroendocrine markers, such as heart rate variability [89], electrodermal activity [90], and cortisol levels [91], with EEG could further clarify the relationship between autonomic arousal and cortical oscillations.

Despite its potential, multimodal imaging presents practical challenges. Simultaneous EEG-fMRI is expensive and sensitive to motion artifacts, and aligning EEG’s fast temporal resolution with fMRI’s slower BOLD signals requires sophisticated data processing. Adequately powered studies require large-scale multicenter collaborations, and multimodal integration requires specialized technical expertise.

Nevertheless, combining EEG with other neuroimaging modalities has the potential to create a comprehensive model of alexithymia’s disrupted neural circuitry. Linking electrophysiological insights with structural and functional imaging—alongside psychophysiological markers—can provide a multidimensional understanding of how emotional processing unfolds in real time and how these mechanisms break down in alexithymia.

### 6.5. Intervention Studies and Clinical Translation

Despite extensive EEG research on alexithymia, few studies have evaluated how these neural markers change after targeted interventions or how they might guide clinical practice. Most work remains cross-sectional, emphasizing group-level differences rather than treatment efficacy. Addressing right-hemisphere overactivation—a frequent EEG finding—is a promising avenue: neurofeedback may help modulate excessive alpha or theta activity in the right parietal or right frontal regions, potentially restoring hemispheric balance [92]. For reduced interhemispheric coherence, interventions that boost cross-hemispheric communication (e.g., eye movement desensitization and reprocessing [93] or transcranial direct current stimulation [94]) may normalize functional connectivity.

Combining EEG monitoring with psychotherapy offers additional benefits. Mindfulness-based interventions improve interoceptive awareness [95,96], and emotion-focused therapies could benefit from ongoing EEG feedback to track alpha desynchronization or theta shifts as emotional self-awareness improves. Tailoring therapy based on specific alexithymia subscales is another strategy: individuals with high Difficulties Identifying Feelings (DIF) might benefit more from body-oriented techniques [97], while those with Difficulties Describing Feelings (DDF) might respond better to language-based approaches [98].

Longitudinal EEG monitoring—e.g., at baseline, mid-treatment, and post-treatment—could clarify whether changes in alpha, theta, or gamma power align with improved emotional functioning, supported by behavioral measures and complementary modalities like heart rate variability (HRV) or fMRI. However, cost, equipment demands, and limited EEG expertise in many clinical settings present practical barriers. Developing portable systems and adaptable protocols could help integrate EEG-based interventions into standard care, ultimately improving emotional awareness and quality of life in individuals with alexithymia.

### 6.6. Refinement of Alexithymia Subtypes

Although alexithymia is often conceptualized as a single construct, emerging research indicates that it comprises multiple subdimensions, each potentially underpinned by distinct neural mechanisms. The Toronto Alexithymia Scale (TAS)—the most widely used self-report measure—encompasses three subscales: Difficulties Identifying Feelings (DIF), Difficulties Describing Feelings (DDF), and Externally Oriented Thinking (EOT) [99]. DIF may be linked to heightened autonomic arousal and right-hemisphere dominance, reflecting strong emotional reactivity but impaired emotion labeling. In contrast, DDF appears to be related to weak interhemispheric connectivity—particularly between the left-hemisphere language areas and limbic structures—making it difficult to express emotions verbally. EOT, which directs attention outward, may manifest as alpha desynchronization in the parietal regions, indicating robust engagement with external stimuli but limited frontal-limbic integration.

Building on these subscales, researchers have proposed “affective” and “cognitive” subtypes of alexithymia [100]. Individuals with the affective subtype display intense but poorly identified emotional experiences (high DIF), often accompanied by increased alpha or theta activity in right-hemisphere regions, whereas the cognitive subtype shows reduced emotional reactivity and verbal-processing deficits (high DDF/EOT), potentially reflecting diminished left-hemisphere connectivity. Future work could apply cluster analysis to TAS subscale profiles and test each group with EEG paradigms tailored to their hypothesized deficits: emotion-labeling tasks for those with affective alexithymia and more cognitively oriented tasks for the cognitive subtype.

Clinically, a subtype-based approach can refine interventions. Individuals scoring high on DIF might benefit most from mindfulness and biofeedback to enhance interoceptive awareness and regulate autonomic arousal [101,102]. Those with pronounced DDF may require explicit emotional language training or expressive writing to bolster the left-hemisphere networks that facilitate emotion labeling. Finally, a high EOT suggests the need for methods—such as guided imagery or structured self-reflection—that redirect attention inward. By matching interventions to an individual’s specific profile, clinicians can target the precise neural disruptions underlying alexithymia, potentially improving emotional awareness, regulation, and the overall quality of life.

## 7. Conclusions

Alexithymia, which has long been described as a difficulty in recognizing and articulating emotions, manifests in clear and measurable EEG signatures that reveal disruptions in both cortical activation and network-level connectivity. The studies surveyed in this review converge on several key themes. First, consistent right-hemisphere dominance and impaired interhemispheric integration emerge as central features, indicating a mismatch between heightened or dysregulated arousal responses and underused verbal-processing mechanisms. Second, although subjective self-reports often fail to distinguish individuals with alexithymia from controls (e.g., in emotional intensity ratings), EEG findings have revealed that individuals with alexithymia process emotional stimuli in qualitatively distinct ways. Specifically, amplified alpha desynchronization, increased theta synchronization, and reduced gamma phase coherence collectively highlight atypical arousal and integrative deficits.

A crucial insight is that these neural signatures do not necessarily indicate the total absence of emotional experience. Rather, individuals with alexithymia appear to register emotional stimuli but struggle with the labeling, modulation, and integration required for nuanced emotional awareness. This underscores the heterogeneity of alexithymic presentations: some individuals may exhibit strong affective reactivity yet lack the means to verbally characterize these states, while others show overall dampened emotional engagement accompanied by limited verbalization skills.

From a mechanistic standpoint, right-hemisphere hyper-responsiveness—combined with impaired or imbalanced engagement from left-hemisphere language and regulatory networks—illustrates how deficits in top-down control may exacerbate bottom-up emotional overload. Reduced coherence within and between hemispheres further suggests a fundamental breakdown in the neural infrastructure that supports flexible shifts between emotional arousal and cognitive evaluation. In higher-frequency domains, decreased gamma activity and reduced gamma phase synchrony indicate a shortfall in the binding mechanisms that help create cohesive emotional representations.

These findings have significant implications for future research and clinical practice. Standardizing EEG protocols, enlarging and diversifying participant samples, and employing longitudinal, multimodal studies will be key to refining our understanding of how—and when—these atypical neural profiles develop. Aligning EEG metrics with specific alexithymia subtypes (e.g., “affective” vs. “cognitive” profiles) could enable the development of tailored interventions, including targeted neurofeedback, mindfulness-based approaches, and explicit emotion-labeling techniques. Ultimately, clarifying the neural circuitry underlying alexithymia offers a pathway toward more personalized and effective interventions, with the promise of helping individuals cultivate the emotional insight and regulation skills they currently lack.

## Figures and Tables

**Table 1:** Summary of the included EEG studies on alexithymia.

Study	Aim/Focus	Participants	EEG Method	Emotional/Resting Paradigm	Key Findings
[35]	Investigate how alexithymia influences cortical activity during emotional stimulation, focusing on interhemispheric asymmetry and right-hemisphere involvement.	44 right-handed university students (17 with TAS ≥ 70, 27 with TAS ≤ 62), matched for sex, ages 18–26.	62-channel EEG Spectral power in theta, alpha, beta, gamma	Eight film clips evoking happiness, anger, disgust, fear, and stress; two neutral clips used for baseline	EEG: Alexithymics showed heightened right-hemisphere activity (theta ↑, alpha ↓) during emotional stimuli. Behavior: No significant differences in subjective emotional intensity between groups.
[36]	Examine how alexithymia affects hemispheric asymmetry in EEG spectral power while viewing emotional film clips.	44 university students (17 with TAS > 74, 27 with TAS < 62), matched for age and sex.	62-channel EEG Spectral power (theta-2, alpha-1, alpha-2)	Ten film clips inducing relaxation, joy, sexual arousal, anger, fear, disgust, sadness, and stress, plus two neutral clips	EEG: Marked right-hemisphere dominance in the alexithymic group, with alpha-2 desynchronization in right centroparietal regions. Behavior: No group differences in self-reported emotional intensity.
[37]	Investigate resting-state DMN activity in alexithymia, focusing on alpha power and functional connectivity, controlling for general psychopathology.	36 adults: 18 alexithymic (TAS-20 ≥ 58) and 18 controls (TAS-20 ≤ 45), matched for age, sex, and socio-demographic variables.	19-channel EEG (10–20 system) eLORETA source localization Lagged phase synchronization	Five-minute resting-state recording (eyes closed)	EEG: Reduced alpha power in right PCC in the alexithymic group; decreased alpha connectivity in right ACC–PCC, frontal–PCC, and parietal–temporal. Psychopathology: These connectivity disruptions remained significant after controlling for GSI (SCL-90-R).
[38]	Examine gamma band oscillations and phase synchrony during emotional processing in individuals with alexithymia and in those without alexithymia.	24 right-handed participants (12 alexithymic with TAS-20 > 61; 12 controls with TAS-20 < 51), no psychiatric or neurological disorders.	EEG with emphasis on gamma band (40–50 Hz) Time-frequency analysis	160 images from IAPS (80 negative, 80 neutral), culturally adapted for Japanese participants	EEG: Non-alexithymic group showed ↑ gamma power & phase synchrony for negative vs. neutral images; alexithymics did not show increased gamma activity. Behavior: Both groups judged negative images as more negative, with no RT differences.
[39]	Investigate differences in EEG coherence and asymmetry in individuals with alexithymia and those without alexithymia, focusing on inter- and intrahemispheric connectivity.	130 right-handed female nurses; top 10% TAS-20K (n = 13) vs. bottom 10% (n = 13).	16-channel EEG (10–20 system) Coherence analysis in delta, theta, alpha, beta	Resting EEG assessment	EEG: Alexithymics showed lower interhemispheric coherence (F3-F4, C3-C4) in theta1, and lower intrahemispheric coherence (fronto-occipital, parieto-central) primarily in the left hemisphere.
[40]	Assess whether individuals with alexithymia show reduced right-frontal and left-hemisphere interaction in emotional and cognitive processing.	20 psychology students (10 high-alexithymic, 10 low-alexithymic), screened via Bermond-Vorst-Alexithymia-Questionnaire (BVAQ). All right-handed.	EEG power (alpha, beta) and coherence. Partial multiple coherence analysis	Viewing three video types: neutral (control), symbolic emotional, and blatant emotional	EEG: Reduced interhemispheric coherence between the right frontal lobe and left hemisphere in alexithymics, independent of emotional film type. Behavior: Emotional clips reduced alpha power at parietal leads in both groups.
[41]	Explore whether frontal alpha asymmetry (FAS) is associated with alexithymia and could serve as a biomarker for emotional dysregulation in BPD.	76 females: 37 with BPD (DSM-5 criteria), 39 healthy controls, matched for age and IQ.	32-channel resting-state EEG FAS calculated from alpha power (8–13 Hz) in left vs. right frontal	Resting-state EEG (eyes closed)	EEG: No significant group-level FAS differences between BPD and controls. However, in BPD patients, FAS at F8-F7 correlated positively with total TAS-20 scores. The relationship was moderated by group and not explained by depression or general psychopathology.
[42]	Investigate theta-band synchronization deficits in individuals with alexithymia during emotional picture viewing and differences in early event-related theta responses.	41 right-handed adults (20 alexithymic, 21 non-alexithymic), screened by TAS.	EEG analysis of theta-1 (4–6 Hz) and theta-2 (6–8 Hz) event-related synchronization	Viewing neutral, pleasant, and unpleasant images (IAPS)	EEG: Alexithymics had reduced/delayed theta synchronization in left anterior regions for emotional stimuli (0–200 ms → 400–600 ms peak), and enhanced right anterior theta for unpleasant stimuli. Behavior: Altered early emotional appraisal.

Abbreviations: TAS: Toronto Alexithymia Scale, BPD: Borderline Personality Disorder, DMN: Default Mode Network, PCC: Posterior Cingulate Cortex, ACC: Anterior Cingulate Cortex, eLORETA: exact Low Resolution Electromagnetic Tomography, FAS: Frontal Alpha Asymmetry, IAPS: International Affective Picture System, ERS: Event-Related Synchronization.

## Data Availability

No new data were created or analyzed during this study. Data sharing is not applicable to this article.
